# Preparation and Antioxidant Activity of Ethyl-Linked Anthocyanin-Flavanol Pigments from Model Wine Solutions

**DOI:** 10.3390/molecules23051066

**Published:** 2018-05-03

**Authors:** Lingxi Li, Minna Zhang, Shuting Zhang, Yan Cui, Baoshan Sun

**Affiliations:** 1 School of Pharmacy, Shenyang Pharmaceutical University, Shenyang 110016, China; lingxilee@163.com; 2 School of Functional Food and Wine, Shenyang Pharmaceutical University, Shenyang 110016, China; zhangshuting@syphu.edu.cn (S.Z.); cuiyan@syphu.edu.cn (Y.C.); 3 School of Traditional Chinese Materia Medica, Shenyang Pharmaceutical University, Shenyang 110016, China; 107000305@syphu.edu.cn; 4 Pólo Dois Portos, Instituto National de Investigação Agrária e Veterinária, I.P., Quinta da Almoinha, Dois Portos 2565–191, Portugal

**Keywords:** anthocyanin, antioxidant activity, ethyl-linked anthocyanin-flavanol pigment

## Abstract

Anthocyanin-flavanol pigments, formed during red wine fermentation and storage by condensation reactions between anthocyanins and flavanols (monomers, oligomers, and polymers), are one of the major groups of polyphenols in aged red wine. However, knowledge of their biological activities is lacking. This is probably due to the structural diversity and complexity of these molecules, which makes the large-scale separation and isolation of the individual compounds very difficult, thus restricting their further study. In this study, anthocyanins (i.e., malvidin-3-glucoside, cyanidin-3-glucoside, and peonidin-3-glucoside) and (–)-epicatechin were first isolated at a preparative scale by high-speed counter-current chromatography. The condensation reaction between each of the isolated anthocyanins and (–)-epicatechin, mediated by acetaldehyde, was conducted in model wine solutions to obtain ethyl-linked anthocyanin-flavanol pigments. The effects of pH, molar ratio, and temperature on the reaction rate were investigated, and the reaction conditions of pH 1.7, molar ratio 1:6:10 (anthocyanin/(–)-epicatechin/acetaldehyde), and reaction temperature of 35 °C were identified as optimal for conversion of anthocyanins to ethyl-linked anthocyanin-flavanol pigments. Six ethyl-linked anthocyanin-flavanol pigments were isolated in larger quantities and collected under optimal reaction conditions, and their chemical structures were identified by HPLC-QTOF-MS and ECD analyses. Furthermore, DPPH, ABTS, and FRAP assays indicate that ethyl-linked anthocyanin-flavanol pigments show stronger antioxidant activities than their precursor anthocyanins.

## 1. Introduction

Grape and wine polyphenols have attracted considerable attention among the international scientific community during the last four decades, not only for their contributions to the quality of wines, including sensory properties (color, flavor, astringency, and bitterness) [1,2,3,4] and aging behavior, but especially for their potential beneficial health effects related to their protective action against coronary heart disease and oxygen free-radical scavenger capacity [5,6,7,8,9,10,11].

Red grape polyphenols essentially consist of anthocyanins (mainly malvidin-3-glucoside, cyanidin-3-glucoside, and peonidin-3-glucoside), flavanols (monomeric (+)-catechin and (–)-epicatechin, and their oligomers and polymers), and small amounts of other phenolics such as phenolic acids, resveratrol and its derivatives, flavonols, flavanonols, and flavones. Red wine polyphenols include both grape polyphenols (anthocyanins and flavanols) and new phenolic products formed from them during the winemaking process (anthocyanin-derived pigments and polymeric flavanols) [12]. Anthocyanin-derived pigments are formed essentially by labile anthocyanins and tend to react with flavanols during the wine fermentation and aging processes [13,14]. 

Studies have shown that anthocyanin contents increase sharply after crushing the grapes during winemaking and reach their maximum within 2 to 3 days of maceration/alcoholic fermentation, but then decrease gradually [15,16]. In the late period of alcohol fermentation, anthocyanins react with flavanols to form anthocyanin-flavanol pigments [17,18]. 

There are two acknowledged mechanisms for the formation of new anthocyanin-flavanol pigments [1,19,20]. One is the direct condensation reaction between anthocyanins (A) and flavanols (F), which forms the direct condensation products, including A^+^-F or F-A^+^ adduct [21]. The other is the indirect condensation reaction between anthocyanins and flavanols mediated by an aldehyde linkage [22,23]. These products arise through nucleophilic addition of the flavanol to a protonated acetaldehyde, giving a new carbocation intermediate which undergoes nucleophilic addition of an anthocyanin in the hemiketal form to ultimately produce ethyl-linked F-Et-A adducts [24]. Indirect condensation reaction occurs quickly and is the main and common polymerization reaction in wine [17,20,25].

Anthocyanin-flavanol pigments from the condensation reaction between anthocyanins and flavanols play an important role in wine color stabilization and are the major contributor to the color of aged wine [26]. The color of young red wine is bright and usually appears violet or ruby red, which is generated solely from anthocyanins. With the formation of anthocyanin-flavanol pigments, red wine acquires an increasingly deep color, appearing brick-red and even red-brown [27].

Condensation reactions lead to the rapid decrease of anthocyanin content, but the color of red wine remains relatively stable because anthocyanin-flavanol pigments are better able to retain their color than anthocyanins under the same pH and SO_2_ conditions [28]. Acetaldehyde, as a byproduct of yeast fermentation that forms through the oxidation of ethanol, can increase the condensation reaction rate of anthocyanins [23,29]. 

Earlier work reported that pigmented complex in red wine account for up to 50% of color density within the first year, and up to 90% after wine aging [30]. Eglinton et al. [18] indicated that pigmented polymers in red wine form during the period of alcohol fermentation. Sun and Spranger [31] reported that during the storage of young red wines, the concentrations of anthocyanins and flavanols decrease significantly while total polyphenols remain stable, suggesting that the majority of anthocyanins and flavanols are transformed to their condensed forms. Quantitatively, anthocyanin-flavanol pigments are one of the major groups of polyphenols in aged red wines [12].

It is well known that moderate consumption of red wine may have beneficial effects, such as protection against certain cancers, improved mental health, and enhanced heart health [32,33,34,35], and that the key compounds responsible for these beneficial effects are polyphenols [11,36,37]. Nevertheless, the available data on the biological activities of red wine polyphenols are limited to those of anthocyanins and flavanols [7,38], resveratrol [39], phenolic acids [40], and flavonols [41]. Indeed, anthocyanins and flavanols are the two major groups of polyphenols in red grapes and in some very young red wines but are present at very low concentrations in aged red wines. For the latter, the major polyphenols (from a quantitative perspective) are polymeric polyphenols, including direct and indirect condensation products between anthocyanins and flavanols [42]. 

However, little is known about the biological properties of the anthocyanin-derived pigments in red wine. This is probably due to the structural diversity and complexity of these compounds, which makes their isolation and further study highly challenging. The objective of the present study was to verify the in vitro antioxidant activity of the ethyl-linked anthocyanin-flavanol pigments formed by indirect condensation reactions between anthocyanins and flavanol that commonly occur during red wine making and storage. For this purpose, we first prepared at large scale the major wine anthocyanins (malvidin-3-glucoside, cyanidin-3-glucoside, and peonidin-3-glucoside), and (–)-epicatechin by high-speed counter-current chromatography (HSCCC) and then performed condensation reactions between anthocyanins and (–)-epicatechin, mediated by acetaldehyde, in a model wine solution. The antioxidant activities of the different ethyl-linked anthocyanin-flavanol pigments isolated under optimized reaction conditions were verified by DPPH, ABTS, and FRAP assays.

## 2. Results and Discussion

### 2.1. HSCCC Separation of Anthocyanins and (–)-Epicatechin

Under optimal conditions, three anthocyanins were successfully separated using HSCCC. The yields of malvidin-3-glucoside, peonidin-3-glucoside, and cyanidin-3-glucoside were 12.12 mg (purity 92.74%), 11.57 mg (purity 91.21%), and 40.33 mg (purity 94.08%), respectively. By further purification, the purities of malvidin-3-glucoside, peonidin-3-glucoside, and cyanidin-3-glucoside reached 98.43%, 97.29%, and 98.9%, respectively.

By one-step HSCCC separation of flavanol under optimized conditions, the product was predominantly (–)-epicatechin, yielding up to 61.28 mg, and >95% purity following purification.

### 2.2. Dynamic Monitoring of Condensation Reactions between Anthocyanins and (–)-Epicatechin

**Figure 1:**
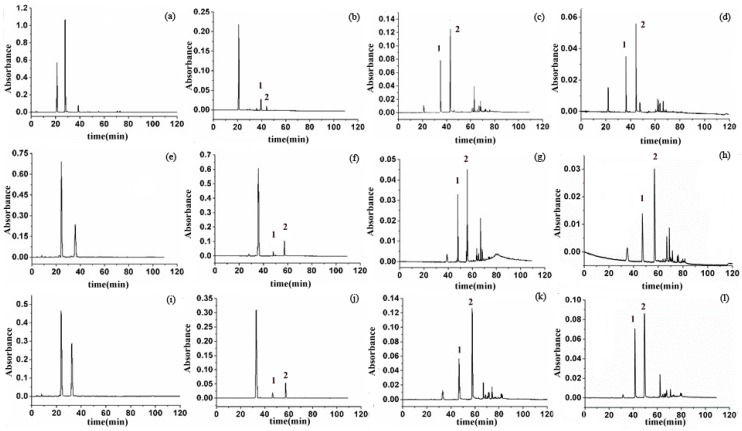
Chromatograms of HPLC analysis of the reaction between cyanidin-3-glucoside and (–)-epicatechin at 0 h (**a**), 2 h (**b**), 224 h (**c**), 336 h (**d**); Chromatograms of HPLC analysis of the reaction between malvidin-3-glucoside and (–)-epicatechin at 0h (**e**), 2 h (**f**), 224 h (**g**), 336 h (**h**); Chromatograms of HPLC analysis of the reaction between peonidin-3-glucoside and (–)-epicatechin at 0 h (**i**), 2 h (**j**), 224 h (**k**), 336 h (**l**).

HPLC-DAD was used for monitoring the changes in concentrations of anthocyanins and ethyl-linked anthocyanin-flavanol pigments during the reaction period. The HPLC chromatograms of the condensation reaction solutions between cyanidin-3-glucoside/malvidin-3-glucoside/peonidin-3-glucoside and (–)-epicatechin at 0, 2, 224, and 336 h in the presence of acetaldehyde at pH 1.7 are presented in Figure 1. Peaks 1 and 2 were the main products of each reaction solution. However, with the reaction process, they tended to break down to form a series of byproducts. The structures of the six main products are illustrated as follows. Table 1 presents the effects of pH, molar ratio, and temperature on the reactivity of anthocyanins towards (–)-epicatechin in the presence of acetaldehyde. The influence of pH on reaction rate constant *K* was significant, with lower pH of reaction medium associated with higher reaction rate. This result was consistent with previous studies [20,22] and might be related to the activity of anthocyanin in the flavylium form under acidic conditions [43]. At the same temperature, the rate constants were 0.0497, 0.0565, and 0.0919 for the reaction between malvidin-3-glucoside and (–)-epicatechin at molar ratios 1:1, 1:3, and 1:6, respectively, at pH 1.7, as compared with 0.0028, 0.0065, and 0.009 for the reaction between malvidin-3-glucoside and (–)-epicatechin at molar ratios 1:1, 1:3, and 1:6, respectively, at pH 3.2. These results indicate that increased (–)-epicatechin molar concentration leads to an increased reaction rate. Since the pH of the wine ranged from 3.2 to 3.5, theoretically, the rate constants of reactions between anthocyanins and (–)-epicatechin in the wine solution are closer to the values obtained at pH 3.2. At 25 °C, pH 1.7, and molar ratio 1:6:10, the conversion of the three anthocyanins to ethyl-linked anthocyanin-flavanol pigments showed differing reaction rate constants, in this sequence: peonidin-3-glucoside > cyanidin-3-glucoside > malvidin-3-glucoside. However, at pH 3.2 representing the pH of red wine, peonidin-3-glucoside showed the highest rate constant and cyanidin-3-glucoside the lowest. The condensation reaction might be influenced by the structure of anthocyanins [44]; the methylation at C-3 position of peonidin-3-glucoside and malvidin-3-glucoside might affect the reaction rate. Meanwhile, the findings for the reaction rate constant *K* might represent the differing contents and stabilities of these three anthocyanins in wine. Peonidin-3-glucoside and malvidin-3-glucoside, which had faster reaction rates, could rapidly react with flavanol and become polymerized into more anthocyanin-flavanol pigments in a stable form in wine, thus contributing more to the wine color. In contrast, cyanidin-3-glucoside, which showed a slower reaction rate, might be easily oxidized and lead to degradation due to the complex variations in conditions during wine aging, therefore contributing less to the wine color than peonidin-3-glucoside and cyanidin-3-glucoside. Similar conclusions were noted by previous researchers [45]. In conclusion, lowering the pH of the reaction medium, increasing the reaction temperature, and increasing the molar ratio of the reactants could increase the reaction rate and produce higher yields of the ethyl-linked anthocyanin-flavanol pigments. Although the reaction rate peaked at 40 °C, the formation and conversion of the main ethyl-linked anthocyanin-flavanol pigments products were both too rapid, thereby hindering their collection. Consequently, reaction conditions pH 1.7, molar ratio 1:6:10, and temperature 35 °C were optimal for large-scale preparation of ethyl-linked anthocyanin-flavanol pigments. 

### 2.3. Structural Identification

The structures of ethyl-linked anthocyanin-flavanol pigments were identified based on molecular ions and MS^2^ fragmentation using HPLC-QTOF-MS and ECD analysis. 

**Figure 2:**
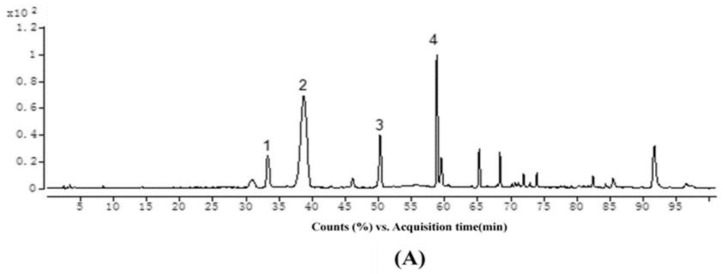

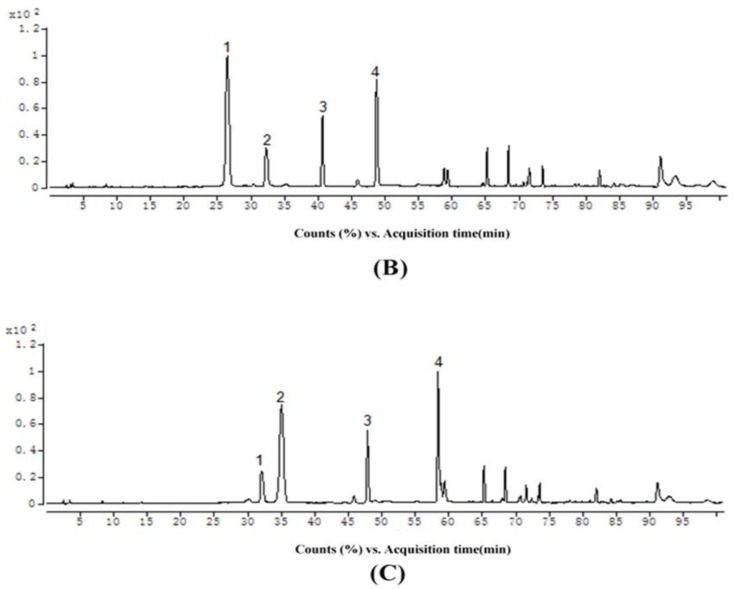
(**A**) Representative TIC chromatogram in positive mode of malvidin-3-glucoside and condensation products: 1. (–)-epicatechin, 2. malvidin-3-glucoside, 3. malvidin-3-glucoside-ethyl-epicatechin (1), 4. malvidin-3-glucoside-ethyl-epicatechin (2); (**B**) Representative TIC chromatogram in positive mode of cyanidin-3-glucoside and condensation products: 1. cyanidin-3-glucoside, 2. (–)-epicatechin, 3. cyanidin-3-glucoside-ethyl-epicatechin (1), 4. cyanidin-3-glucoside-ethyl-epicatechin (2); (**C**) Representative TIC chromatogram in positive mode of peonidin-3-glucoside and condensation products: 1. (–)-epicatechin, 2. peonidin-3-glucoside, 3. peonidin-3-glucoside-ethyl-epicatechin (1), 4. peonidin-3-glucoside-ethyl-epicatechin (2).

**Figure 3:**
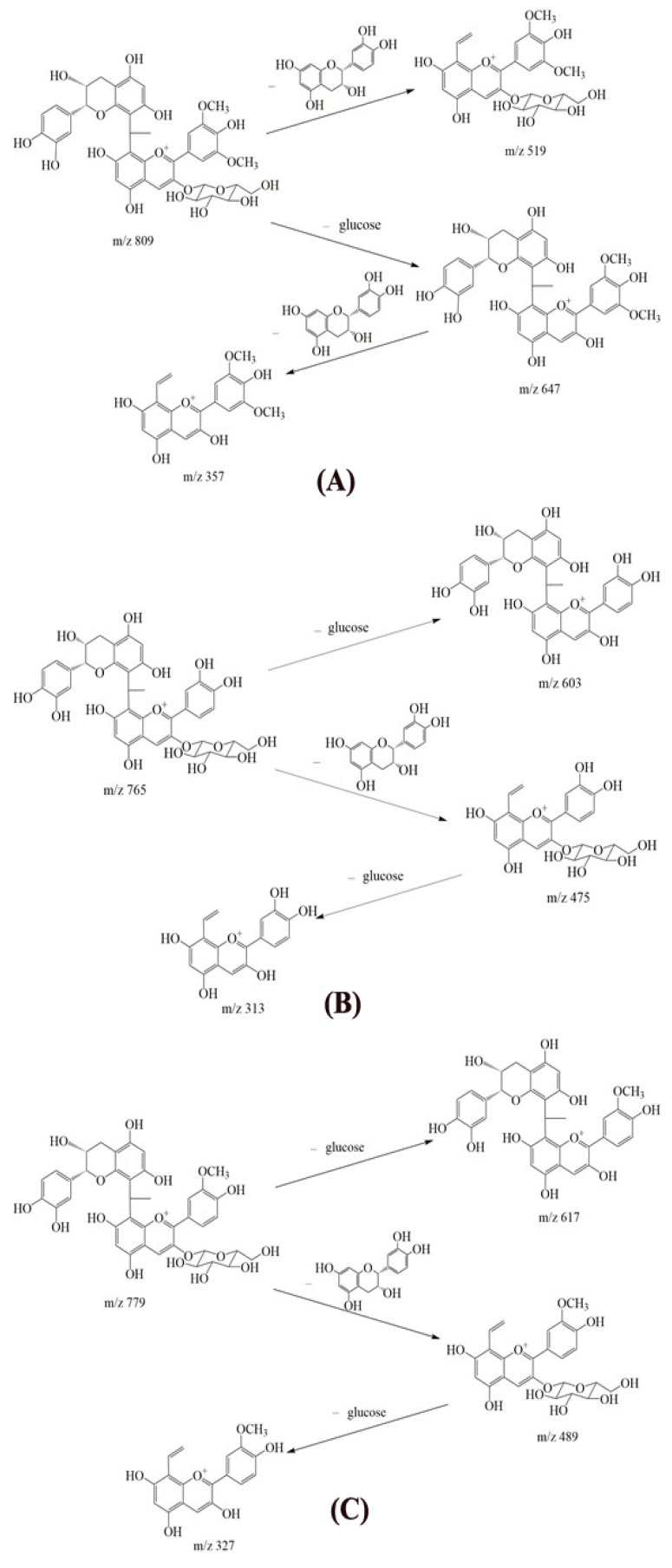
The main fragmentation pathways of condensation products of malvidin-3-glucoside-ethyl-epicatechin (1) (**A**); cyanidin-3-glucoside-ethyl-epicatechin (1) (**B**) and peonidin-3-glucoside-ethyl-epicatechin (1) (**C**).

Representative TIC chromatograms of the reaction mixture are shown in Figure 2. The major ions observed in the MS/MS^2^ spectra are presented in Table 2. In the three reaction mixtures, peaks 3 and 4 (Figure 2) are suggested as the two isomers, showing ions at *m*/*z* 809.2293 and 809.2289, 765.2029 and 765.2025, and 779.2184 and 779.2182, corresponding to the products from condensation reactions between malvidin-3-glucoside, cyanidin-3-glucoside, and peonidin-3-glucoside and (–)-epicatechin mediated by acetaldehyde, respectively. These molecular masses and fragmentation ions were matched well with previously studies [46,47,48]. The C-8 position of the A-ring of either flavanols or anthocyanins was identified as the preferred substitution site for such indirect condensation reactions [30]. Due to the presence of an asymmetric carbon in the ethyl bridge, the two isomers exhibited the same fragmentation patterns. Peak 3 of each reaction mixture was studied as being representative of its fragmentation pattern. Malvidin-3-glucoside-ethyl-epicatechin (1) had an ion with *m*/*z* 809.2293. The ion with *m*/*z* 519.1502 could be derived from the loss of one (–)-epicatechin (290 Da). The ion with *m*/*z* 647.1760 could result from the loss of one glucose moiety (162 Da) and the ion with *m*/*z* 357.0974 might be formed from the further loss of one (–)-epicatechin (290 Da) (Figure 3A). This fragmentation pattern was consistent with that which has been reported in literature [46,49]. Cyanidin-3-glucoside-ethyl-epicatechin (1) had an ion with *m/z* 765.2029. The ion with *m*/*z* 603.1496 could be derived from the loss of one glucose moiety (162 Da). The ion with *m*/*z* 475.1239 could result from the loss of one (–)-epicatechin (290 Da), and the ion with *m*/*z* 313.0713 might be formed from the further loss of one glucose moiety (162 Da) (Figure 3B). Peonidin-3-glucoside-ethyl-epicatechin (1) had ions with *m*/*z* of 779.2184, 617.1655, 489.1395, and 327.0872, indicating a similar fragmentation pattern to that of cyanidin-3-glucoside-ethyl-epicatechin (1), as shown in Figure 3C. These results were in agreement with previous studies [47,50,51]. Molecular ions combined with fragmentation patterns confirmed the identification of the six ethyl-linked anthocyanin-flavanol pigments through comparison with literatures [47,48,49,50,51]. In addition, ions with *m*/*z* of 1125.3240, 1081.2977, and 1095.3126 were detected from three reaction mixtures containing malvidin-3-glucoside, cyanidin-3-glucoside, and peonidin-3-glucoside as the reactant, respectively. It is speculated that these correspond to the structure of one anthocyanin molecule and two (–)-epicatechin molecules linked by two molecule ethyl bridge. However, the ion abundance was weak and their content was too low for further study or separation.

**Figure 4:**
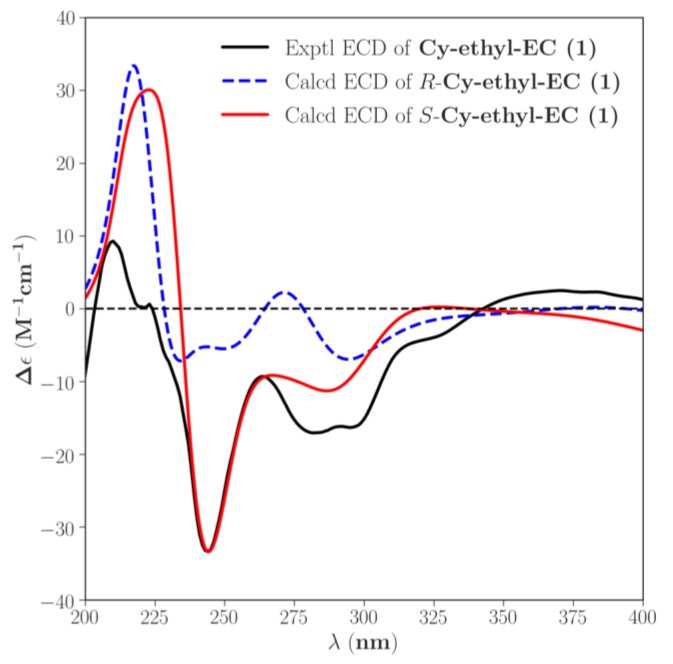
Calculated ECD spectra of configurations *R* and *S* were compared with the experimental.

**Figure 5:**
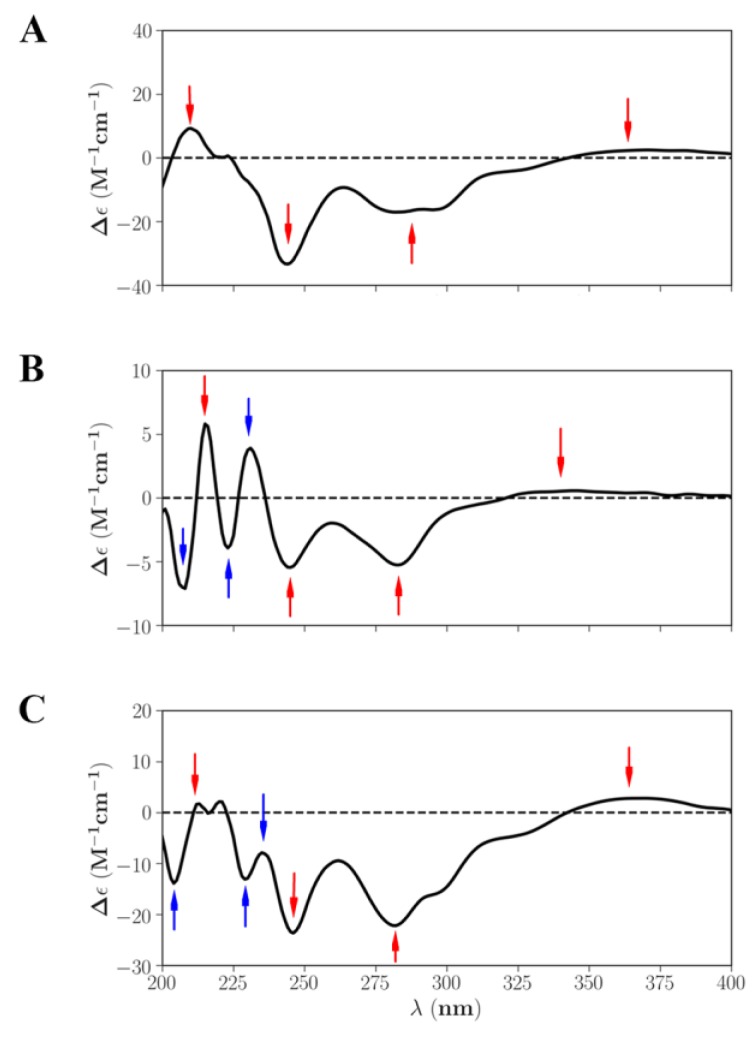
The comparison of CD spectrum of cyanidin-3-glucoside-ethyl-epicatechin (1) (**A**), malvidin-3-glucoside-ethyl-epicatechin (1) (**B**) and peonidin-3-glucoside-ethyl-epicatechin (1) (**C**).

ECD analysis was used to determine the absolute configurations and conformations of chiral molecules. This technique has been widely used for the structural elucidation of natural products. The determination procedure was based on comparing the calculated and experimental ECD spectra [52]. The six ethyl-linked anthocyanin-flavanol pigment molecules contained a chiral carbon atom at the same position, thus ECD calculation was applied to cyanidin-3-glucoside-ethyl-epicatechin (1), and the configurations of the other five molecules were determined by comparison with it. The ECD spectra of cyanidin-3-glucoside-ethyl-epicatechin (1) containing both *R* configuration and *S* configuration were predicted using SpecDis software, and the weighted average method was used to obtain the spectra. The predicted spectra were compared to the experimental spectrum (Figure 4). The results indicate that the ECD spectrum of *S* configuration is consistent with the experimental spectrum, whereas that of the *R* configuration shows obvious differences from the experimental spectrum at 230–250 nm. Therefore, the absolute configuration of cyanidin-3-glucoside-ethyl-epicatechin (1) was identified as *S* configuration. Compared with the experimental spectrum (Figure 5): cyanidin-3-glucoside-ethyl-epicatechin (1), malvidin-3-glucoside-ethyl-epicatechin (1), and peonidin-3-glucoside-ethyl-epicatechin (1) were all identified as *S* configuration, whereas cyanidin-3-glucoside-ethyl-epicatechin (2), malvidin-3-glucoside-ethyl-epicatechin (2), and peonidin-3-glucoside-ethyl-epicatechin (2) were identified as *R* configuration. 

### 2.4. Isolation of Individual Ethyl-Linked Anthocyanin-Flavanol Pigments by Preparative HPLC

**Figure 6:**
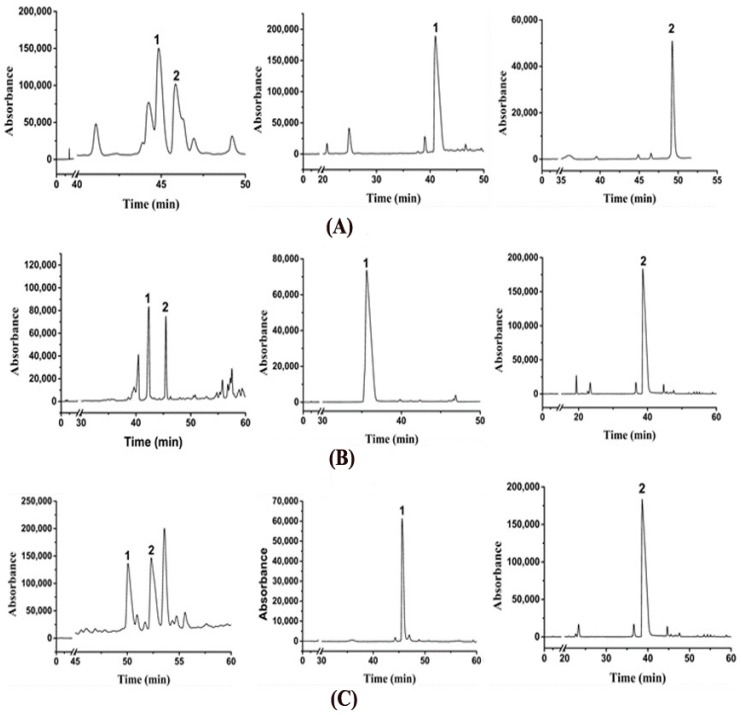
Preparative-HPLC chromatograms of six ethyl-linked anthocyanin-flavanol pigments. (**A**) 1: malvidin-3-glucoside-ethyl-epicatechin (*S*); 2: malvidin-3-glucoside-ethyl-epicatechin (*R*). (**B**) 1: cyanidin-3-glucoside-ethyl-epicatechin (*S*); 2: cyanidin-3-glucoside-ethyl-epicatechin (*R*). (**C**) 1: peonidin-3-glucoside-ethyl-epicatechin (*S*); 2: peonidin-3-glucoside-ethyl-epicatechin (*R*).

Six ethyl-linked anthocyanin-flavanol pigments were isolated and further purified from the reaction solution when the maximum product yields were reached using preparative HPLC under the optimized conditions (Figure 6). Yields of 3.3 mg malvidin-3-glucoside-ethyl-epicatechin (*S*), 4.1 mg malvidin-3-glucoside-ethyl-epicatechin (*R*), 2.2 mg cyanidin-3-glucoside-ethyl-epicatechin (*S*), 6.5 mg cyanidin-3-glucoside-ethyl-epicatechin (*R*), 3.3 mg peonidin-3-glucoside-ethyl-epicatechin (*S*), and 5.1 mg peonidin-3-glucoside-ethyl-epicatechin (*R*) were obtained from conversion of 20 mg malvidin-3-glucoside, cyanidin-3-glucoside, and peonidin-3-glucoside, respectively. The purities of all the products exceeded 95%. The possible impurities in each compound were composed of its isomers or trace amounts of other pigments [44] that had similar properties with it. Therefore, sufficient quantities of individual, high-purity ethyl-linked anthocyanin-flavanol pigments were obtained to provide material guarantee for the study of antioxidant activity.

### 2.5. Antioxidant Activity

Many studies have shown that flavanols and anthocyanins possess strong antioxidant activities in vitro and in vivo [7,53,54,55,56]. However, there are presently few studies on the antioxidant activities of ethyl-linked anthocyanin-flavanol pigments. For determination of six individual ethyl-linked anthocyanin-flavanol pigments as well as their precursor anthocyanins and (–)-epicatechin, three different assay methods (i.e., DPPH, ABTS, and FRAP) were used and the results are presented in Table 3. 

The scavenging capacities of ethyl-linked anthocyanin-flavanol pigments, anthocyanins, and (–)-epicatechin on DPPH· were expressed as EC50 values with two common antioxidants (Vc and Trolox) as controls. The EC50 values of the three anthocyanins, (–)-epicatechin, and six ethyl-linked anthocyanin-flavanol pigments were between 80 μmol/L and 190 μmol/L, much less than the 921 and 1030 μmol/L of Trolox and Vc. Thus, anthocyanins, (–)-epicatechin, and the six ethyl-linked anthocyanin-flavanol pigments all exhibited stronger activities for radical scavenging than Vc and Trolox. The antioxidant abilities were found to decrease in this sequence: ethyl-linked anthocyanin-flavanol pigments > (–)-epicatechin > anthocyanins. Cyanidin-3-glucoside showed the highest antioxidant activity among the three anthocyanins, followed by malvidin-3-glucoside and peonidin-3-glucoside with no significant difference. Comparison of EC50 values among the six ethyl-linked anthocyanin-flavanol pigments demonstrated that cyanidin-3-glucoside-ethyl-epicatechin which was polymerized from cyanidin-3-glucoside possessed better antioxidant capacities, and that there was no significant difference between the isomers of the ethyl-linked anthocyanin-flavanol pigments. 

ABTS assays showed similar results to DPPH assays. Anthocyanins, (–)-epicatechin, and six ethyl-linked anthocyanin-flavanol pigments showed stronger ABTS·^+^ scavenging activities compared with Vc and Trolox. Ethyl-linked anthocyanin-flavanol pigments exhibited more powerful ABTS·^+^ scavenging activity than their precursor anthocyanins and (–)-epicatechin, and cyanidin-3-glucoside-ethyl-epicatechin (*R*) showed the most powerful antioxidant capacity among them. In comparing anthocyanins, cyanidin-3-glucoside exhibited the strongest scavenging capacity, and no significant difference was seen in antioxidant activity between peonidin-3-glucoside and malvidin-3-glucoside. Ethyl-linked anthocyanin-flavanol pigment isomers showed similar antioxidant activities.

The reducing abilities on Fe^3+^-TPTZ (FRAP values) of ethyl-linked anthocyanin-flavanol pigments, anthocyanins, and (–)-epicatechin were calculated by substituting the absorbance values of the analytes into the regression equation of FeSO_4_. The results indicated that anthocyanins, (–)-epicatechin, and ethyl-linked anthocyanin-flavanol pigments are all powerful ferric-reducing antioxidants, compared with the well-known antioxidant Trolox. The ferric ion-reducing activities were found to follow this sequence: ethyl-linked anthocyanin-flavanol pigments > (–)-epicatechin > anthocyanins. Among all the analytes, cyanidin-3-glucoside-ethyl-epicatechin (*R*) showed the highest antioxidant activity and malvidin-3-glucoside the lowest.

The comprehensive results of the three assays demonstrate that anthocyanins, (–)-epicatechin, and ethyl-linked anthocyanin-flavanol pigments all possess strong antioxidant activity. (–)-Epicatechin showed a slightly greater antioxidant capacity than the individual anthocyanins, which was consistent with the results of our previous study [57]. Among the three anthocyanins, cyanidin-3-glucoside presented the highest antioxidant activity followed by peonidin-3-glucoside and malvidin-3-glucoside, in agreement with published studies [58,59]. Previous studies indicated that some pyranoanthocyanins have a higher antioxidant potential than their precursor anthocyanins, whereas others do not [60,61]. In this study, the antioxidant activities of ethyl-linked anthocyanin-flavanol pigments were significantly higher than their precursor anthocyanins, and cyanidin-3-glucoside-ethyl-epicatechin possessed the highest antioxidant activity. In addition, the chiral structure of the ethyl-linked anthocyanin-flavanol pigments showed similar antioxidant capacities. Antioxidant activity was related to the presence of phenolic hydroxyl group. Theoretically, more phenolic hydroxyl groups would be associated with stronger antioxidant activity. The number of phenolic hydroxyl groups in one molecule of ethyl-linked anthocyanin-flavanol pigment was the sum of anthocyanin and (–)-epicatechin, thus ethyl-linked anthocyanin-flavanol pigments showed higher antioxidant activity, which was verified in practice by DPPH, ABTS, and FRAP assays. In addition, hydroxylation and/or methoxylation and their position on the B ring might have an influence on antioxidant capacity [62,63]. Antioxidant activity might be enhanced as a result of the hydroxyl at the C-3′ position on the B ring or subdued due to methylation at the C-3′ and/or C-5′ position [64]. This might be the cause of the differing antioxidant activities of the anthocyanin unit. This is the first reported experimental determination and evaluation of the antioxidant activities of these six ethyl-linked anthocyanin-flavanol pigments, and the findings indicate markedly enhanced antioxidant activity when anthocyanins formed into ethyl-linked anthocyanin-flavanol pigments. This study provides an experimental foundation and theoretical basis for the development and application of ethyl-linked anthocyanin-flavanol pigments as antioxidants.

The correlations of antioxidant activities, determined by ABTS, DPPH, and FRAP assays, were estimated using Pearson’s correlation coefficient (two-tailed). High correlations were found among the assays. The strongest correlation was observed between DPPH and ABTS assays (*r* = 0.991, *P* < 0.001), and the weakest between FRAP and ABTS assays (*r* = −0.882, *P* < 0.001). The DPPH assay showed a strong positive correlation with the ABTS assay, while the FRAP assay was highly negatively correlated with DPPH and ABTS assays, with FRAP and DPPH assays showing the strongest negative correlation (*r* = −0.901, *P* < 0.001). Therefore, the results obtained using the three methods are reliable.

## 3. Materials and Methods

### 3.1. Chemicals and Materials

Acetaldehyde was purchased from Aladdin (Shanghai, China). L-ascorbic acid (V_C_) was purchased from Fluka (Buchs, Switzerland). 2,2-Diphenyl-1-picrylhydrazyl (DPPH), 2,2’-Azino-bis (3-ethylbenzothiazoline-6-sulfonic acid) diammonium salt (ABTS), 2,4,6-Tris(2-pyridyl)-s-triazine (TPTZ), (±)-6-Hydroxy-2,5,7,8-tetramethylchromane-2-carboxylic acid (Trolox) were purchased from Sigma-Aldrich (St. Louis, MO, USA). Blueberry extract was purchased from Tianjin Jianfeng Natural Product R&D Co., Ltd. (Tianjin, China). Red wine extract was provided by Polyphenol Laboratory of Pólo Dois Portos/INIAV (Lisbon, Portugal).

All organic solvents used for HSCCC (analytical grade) and HPLC (chromatographic grade) were purchased from Chemical Branch of Shandong Yuwang Industrial Co., Ltd. (Shandong, China). 

### 3.2. Preparation of Anthocyanins and Flavanols by HSCCC

Malvidin-3-glucoside and peonidin-3-glucoside were isolated from red wine extract by HSCCC (TBE 300B, Tauto Biotechnique Company, Shanghai, China) and combined with preparative HPLC (Waters e2695, Waters, Milford, MA, USA), as described previously in our laboratory [57]. To isolate enough anthocyanins for condensation reactions, red wine extract fermented for 7 days, which is rich in individual anthocyanins, was used. The optimized HSCCC condition was a solvent system consisting of the two phases of methyl tert-butyl ether–*n*-butanol–acetonitrile–water (1-40-1-50, acidified with 0.01% trifluoroacetic acid, *v/v*) with a flow rate of 2 mL/min. One hundred milligrams of red wine extract dissolved in 20 mL lower phase were injected into the apparatus. The HSCCC rotary speed was set at 950 rpm and the detection wavelength was 525 nm. The fractions were collected manually and evaporated to remove organic solvents. The further purification of malvidin-3-glucoside and peonidin-3-glucoside was performed using preparative HPLC, with the mobile phase consisting of 0.2% formic acid-water (solvent A) and 0.2% formic acid-acetonitrile (solvent B). The elution conditions were as follows: malvidin-3-glucoside (12% B, 4 mL/min) and peonidin-3-glucoside (10% B, 3.5 mL/min). The temperature of the column was set at 30 °C.

Blueberry is a rich source of anthocyanins, especially cyanidin-3-glucoside. Thus, cyanidin-3-glucoside was isolated from blueberry extract using HSCCC. A solvent system composed of methyl tert-butyl ether–n-butanol–acetonitrile–water (1-40-1-50, acidified with 0.01% trifluoroacetic acid, *v*/*v*) with a flow rate of 2 mL/min was used as the optimized HSCCC condition. The 20 mL lower phase with 100 mg of blueberry extract was injected through the sample loop. The apparatus was run at 950 rpm and the UV detector was set at 525 nm. The preparative HPLC was used to purify high-purity cyanidin-3-glucoside and the elution condition was 12% B (A: 0.2% formic acid-water; B: acetonitrile) with a flow rate of 4 mL/min and a column temperature of 30 °C.

Typically, procyanidins in cacao beans are mainly composed of (–)-epicatechin structural units. To obtain (–)-epicatechin on a large scale as a reactant for condensation reactions, (–)-epicatechin was separated from cacao-bean phenolic extract using HSCCC based on our previous work [65]. The cacao-bean phenolic extract was prepared according to our previously published procedure [66]. The optimized HSCCC condition was as follows: The two-phase solvent system was *n*-hexane-ethyl acetate-water (1:50:50, *v*/*v*) and flow rate was 3 mL/min. The sample solution was prepared by dissolving 300 mg of the cacao-bean phenolic extract in the 20 mL lower phase. Both the tail-head and head-tail elution modes were used with a rotary speed of 950 rpm and a detection wavelength of 280 nm. (–)-Epicatechin was further purified through preparative HPLC in large scale with water (solvent A) and methanol (solvent B) as the mobile phase with an elution condition of 30% B. The column temperature was set at 30 °C. The flow rate of the mobile phase was fixed at 3 mL/min. 

### 3.3. Model Wine Solution

The model wine solution used for the condensation reactions was composed of 12% ethanol and 5 g/L l-tartaric acid dissolved in water, adjusted to pH 3.2 with 1 mol/L HCl.

### 3.4. Optimization of the Condensation Reaction between Anthocyanins and Flavanols Mediated by Acetaldehyde

Two pH values, 3.2 and 1.7, were chosen for the model solutions and the pH was adjusted by the addition of 1 mol/L HCl or 1 mol/L NaOH. The pH values of 3.2 and 1.7 correspond to the pH of red wine and the pH at which anthocyanins are mainly present in the flavylium form, respectively. At each pH value, the reaction medium was prepared by combining anthocyanins (malvidin-3-glucoside, cyanidin-3-glucoside, and peonidin-3-glucoside), (–)-epicatechin, and acetaldehyde in a molar ratio of 1:6:10 at 25 °C in brown glass vials. The molar ratio of anthocyanins/ (–)-epicatechin (1:6) used in this study corresponded to that in red wine, which is rich in these compounds [66]. Acetaldehyde, which was used as an excess reactant to provide ethyl linkage, was added to the reaction solution according to our previous procedure with slight modifications [67]. Malvidin-3-glcoside/(–)-epicatechin/acetaldehyde molar ratios of 1:3:10 and 1:1:10 were also studied to determine the conditions for optimal utilization of the reactants. The reaction between malvidin-3-glcoside and (–)-epicatechin was studied at reaction temperatures of 30, 35, and 40 °C in to determine the effect of temperature on the reaction rate. A total of 16 reaction systems were established. The reaction products were monitored periodically by HPLC-DAD and ESI-MS under the conditions described below. The optimized reaction conditions were determined from the reaction rates and the maximum yields of the reaction products.

### 3.5. HPLC-DAD Analysis

The HPLC system was used to monitor the reaction products, equipped with a quaternary pump (Waters e2695), a controller (Waters e2695), an autosampler (Waters e2695), and a photodiode array detector (2998 PDA detector) coupled to a data processing computer (Empower^TM^ 2 chromatography data software). The column was Innoval C18 (5 μm, 4.6 × 250 mm) and the temperature was set at 30 °C. The flow rate of the mobile phase was fixed at 0.7 mL/min. Two elution solvents, A (water:formic acid; 98:2, *v*/*v*) and B (water:acetonitrile:formic acid; 68:30:2, *v*/*v*), were used with the gradient elution program as follows: 0 min, 18% B; 42–48 min, 47% B; 78–110 min, 100% B; and re-equilibration of the column for 10 min. The detection wavelength was 525 nm for the detection of anthocyanins and their derivatives and 280 nm for all polyphenols.

### 3.6. Isolation and Purification of Ethyl-Linked Anthocyanin-Flavanol Pigments 

Individual ethyl-linked anthocyanin-flavanol pigments were formed from each of the condensation reactions between anthocyanins and (–)-epicatechin mediated by acetaldehyde under the optimized conditions described above. When the maximal yields of the reaction products were reached in the reaction solution, the ethyl-linked anthocyanin-flavanol pigments were isolated and purified using a Shimadzu LC-20AR module equipped with an SPD-20AV detector coupled to a data processing computer (LabSolutions, Kyoto, Japan). The wavelength was set at 280 nm. The column was YMC-Pack ODS-A (250 × 10 mm, 5 μm) and the temperature was kept at 30 °C. The flow rate of the mobile phase was fixed at 4.0 mL/min. To isolate individual ethyl-linked anthocyanin-flavanol pigments, gradient elution was performed with two solvents, A (water:formic acid; 98:2, *v*/*v*) and B (water:acetonitrile:formic acid; 68:30:2, *v*/*v*), as follows: 0 min, 18% B; 15 min, 47% B; 15–25 min, 47% B; 55 min, 100% B. For further purification of the ethyl-linked anthocyanin-flavanol pigments, isocratic elution was performed with two solvents, A (water:formic acid; 98:2, *v*/*v*) and B (acetonitrile), under chromatographic conditions of 12% B.

### 3.7. MS Analysis

Identification of the compounds formed in the reaction solution was carried out by HPLC-ESI-QTOF-MS/MS (Agilent Technologies, Santa Clara, CA‎, USA) analysis. MS/MS analysis was performed in positive ion mode using the following conditions: mass range recorded was from *m/z* 50–1500; capillary voltage was 3500 V; gas temperature was 300 °C; gas flow was 7 L/min; nebulizer pressure was 35 psi; sheath gas temperature was 325 °C; sheath gas flow 11 L/min.

### 3.8. ECD Analysis

#### 3.8.1. Circular Dichroism (CD) Spectra

CD spectra were recorded in the range of 200–550 nm using a Bio-Logic MOS-450 spectrometer (Bio-Logic, Claix, France) at 25 °C. The path length of the quartz cuvette was 1 cm. The sampling interval was set to 0.5 s.

#### 3.8.2. Conformational Analysis

Conformational analysis was initially performed using Confab [68] with the MMFF94 force field to systematically search for undetermined relative configurations (*R* and *S*) of cyanidin-3-glucoside-ethyl-epicatechin compounds. As the saccharide group was expected to have less of an effect on the ECD spectra, it was removed to simplify the structure in conformational analyses and ECD calculations. Room-temperature equilibrium populations were calculated according to the Boltzmann distribution law (Equation (1)). Conformers with a Boltzmann population over 0.01% were chosen for subsequent Quantum Mechanics (QM) calculations.

(1) $$\frac{N_{i}}{N}=\frac{g_{i}e^{-\frac{E_{i}}{k_{B}T}}}{\sum g_{i}e^{-\frac{E_{i}}{k_{B}T}}}$$

where $N_{i}$ is the number of conformer i with energy $E_{i}$ and degeneracy $g_{i}$ at temperature T, and $k_{B}$ is Boltzmann constant.

#### 3.8.3. ECD Calculation

The theoretical calculations were conducted using Gaussian 09 (Revision D.01. Gaussian Inc., Wallingford, CT, USA). Firstly, conformers were optimized at the PM6 level of theory using a semiempirical method. The conformers with a Boltzmann population lower than 1% were filtered and the remaining conformers were further optimized at the B3LYP/6-311G (d,p) level of theory in methanol using the IEFPCM model. Vibrational frequency analysis confirmed the stable structures. Under the same conditions, the ECD calculation was conducted using time-dependent density functional theory (TD-DFT). Rotatory strengths for a total of 100 excited states were calculated. The ECD spectrum was simulated in SpecDis 1.64 [69] by overlapping Gaussian functions for each transition according to Equation (2).

(2) $$\Delta \varepsilon \left(E\right)=\frac{1}{2.297\times 10^{-39}}\times \frac{1}{\sqrt{2\pi \sigma }}\overset{A}{\underset{i}{\sum }}\Delta E_{i}R_{i}e^{-{\left(\frac{E-E_{i}}{2\sigma }\right)}^{2}}$$

where σ represents the width of the band at 1/e height, and $\Delta E_{i}$ and $R_{i}$ are the excitation energies and rotatory strengths for transition i, respectively. 

The *σ* and UV-shift parameters were 0.32 eV and 20 nm, respectively, for *R* configurations, and 0.32 eV and 30 nm, respectively, for *S* configurations.

### 3.9. Antioxidant Activity

The in vitro antioxidant activities of (–)-epicatechin, anthocyanins (malvidin-3-glucoside, cyanidin-3-glucoside, and peonidin-3-glucoside), and ethyl-linked anthocyanin-flavanol pigments were analyzed by three common methods. DPPH and ABTS assays were implemented by measuring free-radical scavenging capacities, whereas the FRAP assay was conducted by evaluating ferric-reducing antioxidant power. 

#### 3.9.1. DPPH Assay

The DPPH assay has been considered a standard and easy colorimetric method for estimating antioxidant properties by assessing the free-radical scavenging capacities of the antioxidants. The scavenging abilities of the samples for DPPH were determined as described in our previous method with slight modifications [65]. Briefly, a DPPH solution was diluted with methanol to obtain an absorbance of 0.74 (±0.02) at 517 nm. Then, 5 μL of the sample solution or standard solution of Vc and Trolox at various concentrations and 200 μL of DPPH solution were added to a 96-well microplate (Corning, NY, USA). The absorbance of the reaction mixture at 517 nm was recorded using a microplate reader (Tecan Infinite M200 Pro, Männedorf, Switzerland) at the steady state reached after 100 min of reaction at room temperature in the dark, using methanol as a blank reference. The scavenging ability for DPPH radicals was calculated using Equation (3):

(3) Scavenging rate (%)= (A0−Ai) × 100

where A0 and Ai are the absorbance of the control and the sample, respectively.

The antioxidant activity was expressed as EC50, defined as the amount of antioxidant needed to decrease the initial free-radical concentration by 50%. The EC50 value can be obtained from the dose–response curve using regression analysis. The calculation was performed using SPSS software (Version 22.0, Chicago, IL, USA).

#### 3.9.2. ABTS Assay

Antioxidant activities can be evaluated by measuring the ability to scavenge the ABTS·^+^ free radical according to our previous method [65] with some modifications. An ABTS stock solution was obtained by dissolving ABTS in a phosphate buffered saline (PBS, pH 7.4) solution to a 7 mM concentration. Equal amounts of ABTS stock solution and 2.45 mM potassium persulfate were mixed and then allowed to react in the dark at room temperature for 12–16 h to produce ABTS radical cations (ABTS·^+^). The ABTS·^+^ solution was then diluted with PBS to obtain an absorbance of 0.74 ± 0.03 at 734 nm before use. Then, 200 μL of ABTS·^+^ solution was added to each well of a 96-well microplate together with 10 μL of sample solution or standard solution at various concentrations. After reacting at room temperature for 240 min, the absorbance was recorded at 734 nm against a blank reference of PBS using microplate reader described above. The ABTS·^+^ radical cation scavenging activity was calculated using Equation (3) and the EC50 value was obtained as described above.

#### 3.9.3. FRAP Assay

The ferric-reducing antioxidant power (FRAP) assay, based on the reduction of Fe^3+^-TPTZ to blue-colored Fe^2+^-TPTZ, was conducted based on the procedure described in our previous work [65] with slight modifications. Briefly, the FRAP stock solution was composed of 300 mM acetate buffer (pH 3.6), 10 mM TPTZ solution in 40 mM HCl, and 20 mM FeCl_3_·6H_2_O solution. A fresh working solution was prepared by mixing these three solutions at a ratio of 10:1:1 at 37 °C. Sample (5 μL) or standard solutions at various concentrations were allowed to react with 180 μL of the working solution at 37 °C for 390 min in a 96-well microplate. Then, the absorbance was recorded at 593 nm, against a reagent blank. The FRAP value was expressed as μM FeSO_4_/μM sample under the same absorbance.

### 3.10. Statistical Analysis

All experiments were performed in triplicate and results are expressed as means ± standard deviation (SD). The comparison of means was determined by one-way analysis of variance (ANOVA) followed by Duncan’s multiple range tests. Correlations among data obtained were calculated using Pearson’s correlation coefficient (r). All statistical analyses were performed using SPSS (Version 22.0, Chicago, IL, USA).

## 4. Conclusions

Condensation reactions between three anthocyanins (malvidin-3-glucoside, cyanidin-3-glucoside, and peonidin-3-glucoside) and (–)-epicatechin mediated by acetaldehyde were implemented in a model wine solution. The optimized reaction conditions for ethyl-linked anthocyanin-flavanol pigments were obtained. Six individual ethyl-linked anthocyanin-flavanol pigments were isolated from the reaction mixture and their structures were identified by HPLC-QTOF-MS and ECD analyses. Furthermore, all six ethyl-linked anthocyanin-flavanol pigments showed higher antioxidant activities than anthocyanins, and the ethyl-linked anthocyanin-flavanol pigments containing cyanidin-3-glucoside unit exhibited the highest antioxidant properties. The effect of configuration on the antioxidant activities of the ethyl-linked anthocyanin-flavanol pigments was not significant.

## Figures and Tables

**Table 1:** Rate constant of reaction between anthocyanin and (–)-epicatechin mediated by acetaldehyde at different reaction conditions in model wine solution.

Reactants (anthocyanin + EC)	Reaction Condition (Temperature, Molar Ratio (anthocyanin/EC/acetaldehyde))			
	pH = 1.7	Rate Constant *K* (h^−1^)	pH = 3.2	Rate Constant *K* (h^−1^)
Mv + EC	25 °C, 1:1:10	0.0497	25 °C, 1:1:10	0.0028
	25 °C, 1:3:10	0.0565	25 °C, 1:3:10	0.0065
	25 °C, 1:6:10	0.0919	25 °C, 1:6:10	0.0090
	30 °C, 1:6:10	0.1763	30 °C, 1:6:10	0.0191
	35 °C, 1:6:10	0.3207	35 °C, 1:6:10	0.0413
	40 °C, 1:6:10	0.7693	40 °C, 1:6:10	0.0910
Cy + EC	25 °C, 1:6:10	0.1005	25 °C, 1:6:10	0.0066
Pn + EC	25 °C, 1:6:10	0.1630	25 °C, 1:6:10	0.0101

Abbreviations: Cy, cyanidin-3-glucoside; EC, (–)-epicatechin; Mv, malvidin-3-glucoside; Pn, peonidin-3-glucoside.

**Table 2:** Anthocyanins and condensation reaction products detected by ESI-MS^2^.

Sample	No.	Compound	[M]^+^	MS^2^ Product Ions (*m*/*z*)
Mv + EC	1	Mv	493.1344	331.0812
	2	Mv-ethyl-EC (1)	809.2293	647.1760, 519.1502, 357.0974
	3	Mv-ethyl-EC (2)	809.2289	647.1763, 519.1497, 357.0976
	4	Mv-ethyl-EC-ethyl-EC	1125.3240	
Cy + EC	1	Cy	449.1082	287.0551
	2	Cy-ethyl-EC (1)	765.2029	603.1496, 475.1239, 313.0713
	3	Cy-ethyl-EC (2)	765.2025	603.1500, 475.1235, 313.0715
	4	Cy-ethyl-EC-ethyl-EC	1081.2977	
Pn + EC	1	Pn	463.1239	301.0707
	2	Pn-ethyl-EC (1)	779.2184	617.1655, 489.1395, 327.0872
	3	Pn-ethyl-EC (2)	779.2182	617.1659, 489.1391, 327.0872
	4	Pn-ethyl-EC-ethyl-EC	1095.3126	

Abbreviations: Cy, cyanidin-3-glucoside; EC, (–)-epicatechin; Mv, malvidin-3-glucoside; Pn, peonidin-3-glucoside.

**Table 3:** Antioxidant activities and linear ranges of (–)-epicatechin, anthocyanins, ethyl-linked anthocyanin-flavanol pigments and antioxidants by DPPH, ABTS, and FRAP assays.

Compounds	DPPH (μmol/L)		ABTS (μmol/L)		FRAP (μmol/L)	
	EC50	Linear Range	EC50	Linear Range	FRAP Value	Linear Range
Pn	189 ± 3 ^c^	24.8–198.5	107 ± 2 ^c^	19.8–148.8	10.3 ± 0.0059 ^g^	24.3–194.8
Mv	190 ± 10 ^c^	24.3–194.8	106 ± 2 ^c^	19.5–146.1	8.9 ± 0.0070 ^h^	24.8–248.1
Cy	170 ± 5 ^d^	48.9–244.6	103 ± 1 ^d^	19.6–146.7	10.8 ± 0.0056 ^f^	12.2–146.7
EC	153 ± 4 ^e^	50.2–301.2	99 ± 1 ^e^	50.2–200.8	11.5 ± 0.0069 ^e^	25.1–200.8
Pn-ethyl-EC (*S*)	130 ± 4 ^f^	24–192	95 ± 2 ^f^	24–192	12.2 ± 0.0045 ^d^	24–192
Pn-ethyl-EC (*R*)	121 ± 2 ^f^	24.5–196.1	90 ± 2 ^g^	24.5–196.1	12.4 ± 0.0079 ^d^	25.6–205
Mv-ethyl-EC (*S*)	132 ± 1 ^f^	25.6–205	86 ± 1 ^h^	25.6–153.8	13.3 ± 0.0069 ^b^	24.8–198
Mv-ethyl-EC (*R*)	135 ± 2 ^f^	24.8–198	87 ± 2 ^g,h^	29.7–118.8	12.9 ± 0.0074 ^c^	12.2–145.8
Cy-ethyl-EC (*S*)	83 ± 2 ^g^	12.2–97.2	79 ± 3 ^i^	24.3–116.7	12.4 ± 0.0061 ^d^	12.2–146
Cy-ethyl-EC (*R*)	80 ± 3 ^g^	12.2–97.3	77 ± 3 ^i^	24.3–116.8	16.5 ± 0.0048 ^a^	12.5–150
V_C_	1030 ± 27 ^a^	125.5–2007.6	469 ± 4 ^a^	200.8–702.6		
Trolox	921 ± 17 ^b^	200.4–1002	447 ± 2 ^b^	200.4–701.4	2.2 ± 0.0049 ^i^	200.4–1002
FeSO_4_						400–2000.1

Abbreviations: Cy, cyanidin-3-glucoside; EC, (–)-epicatechin; Mv, malvidin-3-glucoside; Pn, peonidin-3-glucoside. EC50 values or FRAP values were presented as mean ± standard deviation of three independent experiments (*n* = 3). Superscript (a–i) in a column mean significant differences, *p* < 0.05.
